# Supplementary material for: Gender Differences in Unhealthy Lifestyle Behaviors among Adults with Diabetes in the United States between 1999 and 2018
Source: Int J Environ Res Public Health. 2022 Dec 7;19(24):16412. doi: 10.3390/ijerph192416412 (PMC9778889; doi:10.3390/ijerph192416412)

SUPPLEMENTAL MATERIAL

Table S1: Total participants and adult diabetes patients number in the NHANES survey

Year	Women		Men	
	Number of Adults with Diabetes	Number of Adult Participants	Number of Adults with Diabetes	Number of Adult Participants
1999~2000	289	5082	286	4883
2001~2002	295	5708	330	5331
2003~2004	317	5152	332	4970
2005~2006	306	5268	316	5080
2007~2008	489	5053	509	5096
2009~2010	464	5312	516	5225
2011~2012	441	4900	470	4856
2013~2014	446	5172	473	5003
2015~2016	481	5079	558	4892
2017~2018	516	4697	578	4557
Total	4044	51423	4368	49893

Table S2. Age-standardized proportion of unhealthy lifestyle behaviors among adults with diabetes in 1999-2000, by gender

	Men (N=286)	Women (N =289)	Men vs Women
Age (%)			*P*=0.506
20-39 y	6.40(2.92-9.87)	8.17(4.32-12.03)	
40-59 y	31.61(24.06-39.15)	28.25(20.23-36.26)	
≥60 y	62.00(55.30-68.70)	63.58(55.19-71.97)	
Race/Ethnicity (%)			*P*=0.006
Non-Hispanic White	70.95(61.35-80.55)	55.84(44.31-67.36)	
Non-Hispanic Black	11.16(5.54-16.79)	20.28(12.07-28.49)	
Hispanic	13.60(3.61-23.59)	16.17(3.94-28.40)	
Others	4.28(0.11-8.46)	7.72(1.06-14.37)	
Insurance Status (%)			*P*=0.371
Uninsured	9.87(5.54-14.19)	13.25(6.42-20.09)	
Insured	88.72(83.86-93.58)	84.26(77.31-91.20)	
Employment Status (%)			*P*＜0.001
Unemployed	48.51(40.92-56.10)	24.92(19.82-30.02)	
Employed	51.49(43.90-59.08)	75.08(69.98-80.18)	
Marital Status (%)			*P*＜0.001
Married or living with partner	70.45(59.33-81.58)	41.55(29.01-54.09)	
Never married	3.85(0.50-7.20)	9.94(5.71-14.17)	
Widowed, divorced, separated	13.70(6.87-20.52)	37.69(27.27-48.12)	
Education Status (%)			*P*=0.126
Below High School	32.34(24.03-40.65)	20.64(12.10-29.19)	
High school graduate or GED	26.68(17.90-35.47)	30.78(19.74-41.81)	
Some college or Above	40.71(33.00-48.41)	48.38(40.11-56.65)	
Family Income Status (%)			*P*=0.258
PIR <1.30	24.06(15.17-32.95)	30.55(18.40-42.71)	
PIR: 1.30-3.49	37.18(27.45-46.91)	41.10(34.14-48.06)	
PIR ≥3.50	24.93(19.00-30.87)	13.96(9.88-18.03)	
AHA Secondary Diet Score	36.30(±0.65)	37.54(±1.31)	-1.23(±1.90)
Poor Diet (%)	41.64(36.07-47.22)	35.38(23.77-46.99)	6.27(-6.51-19.04)
PHQ-9 score	-	-	
Depression (%)	-	-	
BMI (kg/m^2)	31.17(±0.60)	32.62(±0.46)	-1.44(±0.76)
Obesity (%)	47.04(38.23-55.84)	55.90(50.54-61.26)	-8.86(-19.09-1.37)
Smoking (%)	22.99(14.13-31.84)	13.47(8.30-18.65)	9.52(-0.66-19.69)
Alcohol Consumption (%)	49.91(37.14-62.69)	32.02(24.94-39.11)	17.89(3.40-32.38)
Insufficient Physical Activity (%)	-	-	
Sedentary Behavior (%)	-	-	

AHA, American Heart Association; BMI, body mass index; GED, General Educational Development; PHQ, Patient Health Questionnaire; PIR, Poverty Impact Ratio. Values are mean for continuous variables and percentage for categorical variables, age-standardized by the age composition ratio of adults with diabetes. Values between brackets indicate 95% CI for constituent ratio. Values between brackets indicate standard deviation (SD) for mean.

Table S3. Age-standardized proportion of unhealthy lifestyle behaviors among adults with diabetes in 2001-2002, by gender

	Men (N =330)	Women (N =295)	Men vs Women
Age (%)			*P*=0.277
20-39 y	6.82(2.11-11.54)	10.03(5.37-14.70)	
40-59 y	39.82(32.95-46.68)	25.71(21.38-30.04)	
≥60 y	53.36(45.62-61.09)	64.25(58.08-70.43)	
Race/Ethnicity (%)			*P*=0.100
Non-Hispanic White	68.30(60.87-75.73)	61.05(52.15-69.95)	
Non-Hispanic Black	11.56(7.15-15.97)	18.10(10.45-25.76)	
Hispanic	13.44(5.11-21.78)	13.81(5.29-22.33)	
Others	6.70(3.22-10.18)	7.03(1.12-12.95)	
Insurance Status (%)			*P*=0.302
Uninsured	9.74(5.70-13.78)	6.68(3.15-10.21)	
Insured	87.75(83.43-92.06)	91.46(87.34-95.58)	
Employment Status (%)			*P*＜0.001
Unemployed	49.15(43.25-55.05)	26.06(21.47-30.66)	
Employed	50.85(44.95-56.75)	73.94(69.34-78.53)	
Marital Status (%)			*P*＜0.001
Married or living with partner	73.21(67.62-78.79)	41.39(31.40-51.37)	
Never married	8.30(4.84-11.76)	11.63(5.85-17.41)	
Widowed, divorced, separated	18.49(13.38-23.60)	46.99(36.90-57.08)	
Education Status (%)			*P*=0.242
Below High School	44.23(37.13-51.34)	38.40(31.56-45.25)	
High school graduate or GED	22.78(16.34-29.23)	25.17(19.10-31.24)	
Some college or Above	32.36(26.60-38.13)	36.43(29.63-43.22)	
Family Income Status (%)			*P*=0.044
PIR <1.30	23.20(17.21-29.20)	30.93(23.75-38.10)	
PIR: 1.30-3.49	33.38(26.10-40.67)	37.77(29.56-45.98)	
PIR ≥3.50	35.19(26.59-43.79)	20.47(14.83-26.11)	
AHA Secondary Diet Score	36.12(±1.44)	40.28(±1.05)	-4.16(±1.48)
Poor Diet (%)	38.02(30.43-45.61)	26.35(19.67-33.03)	11.67(1.64-21.71)
PHQ-9 score	-	-	
Depression (%)	-	-	
BMI (kg/m^2)	31.66(±0.70)	32.29(±0.46)	-0.63(±0.65)
Obesity (%)	44.65(37.12-52.17)	50.39(45.11-55.68)	-5.75(-14.87-3.37)
Smoking (%)	24.99(18.55-31.43)	16.09(11.63-20.56)	8.90(1.12-16.67)
Alcohol Consumption (%)	55.86(47.78-63.95)	35.97(28.81-43.13)	19.89(9.18-30.61)
Insufficient Physical Activity (%)	-	-	
Sedentary Behavior (%)	-	-	

AHA, American Heart Association; BMI, body mass index; GED, General Educational Development; PHQ, Patient Health Questionnaire; PIR, Poverty Impact Ratio. Values are mean for continuous variables and percentage for categorical variables, age-standardized by the age composition ratio of adults with diabetes. Values between brackets indicate 95% CI for constituent ratio. Values between brackets indicate standard deviation (SD) for mean.

TableS4. Age-standardized proportion of unhealthy lifestyle behaviors among adults with diabetes in 2003-2004, by gender

	Men (N =332)	Women (N =317)	Men vs Women
Age (%)			*P*=0.813
20-39 y	7.61(3.17-12.06)	6.90(2.95-10.84)	
40-59 y	33.37(27.62-39.11)	34.87(27.47-42.27)	
≥60 y	59.02(50.89-67.15)	58.23(52.47-64.00)	
Race/Ethnicity (%)			*P*=0.095
Non-Hispanic White	71.96(62.20-81.72)	63.70(52.75-74.66)	
Non-Hispanic Black	10.10(6.82-13.38)	17.07(9.61-24.53)	
Hispanic	11.85(5.10-18.60)	10.49(3.06-17.92)	
Others	6.09(1.65-10.53)	8.74(4.57-12.91)	
Insurance Status (%)			*P*=0.234
Uninsured	8.30(4.98-11.63)	11.55(8.79-14.30)	
Insured	90.88(87.39-94.37)	88.30(85.62-90.98)	
Employment Status (%)			*P*=0.182
Unemployed	39.39(27.86-50.92)	30.80(23.67-37.92)	
Employed	60.61(49.08-72.14)	68.97(61.73-76.20)	
Marital Status (%)			*P*＜0.001
Married or living with partner	73.41(68.42-78.40)	50.99(43.13-58.86)	
Never married	8.41(3.98-12.85)	7.86(3.84-11.88)	
Widowed, divorced, separated	18.01(15.14-20.87)	41.15(33.93-48.36)	
Education Status (%)			*P*=0.014
Below High School	52.51(46.52-58.50)	38.00(30.80-45.20)	
High school graduate or GED	20.33(15.27-25.39)	28.92(23.43-34.41)	
Some college or Above	26.99(21.69-32.29)	33.08(25.44-40.72)	
Family Income Status (%)			*P*＜0.001
PIR <1.30	16.41(12.53-20.28)	27.75(21.60-33.90)	
PIR: 1.30-3.49	38.02(30.11-45.94)	40.36(34.08-46.65)	
PIR ≥3.50	39.46(31.70-47.23)	22.64(18.07-27.20)	
AHA Secondary Diet Score	37.59(±0.89)	38.92(±0.73)	-1.32(±1.21)
Poor Diet (%)	35.84(27.57-44.11)	28.05(20.32-35.78)	7.79(-3.44-19.02)
PHQ-9 score	-	-	
Depression (%)	-	-	
BMI (kg/m^2)	30.84(±0.50)	32.31(±0.69)	-1.47(-±0.73)
Obesity (%)	51.35(44.91-57.80)	54.35(45.10-63.60)	-2.99(-14.18-8.19)
Smoking (%)	28.02(23.45-32.58)	16.22(12.10-20.34)	11.79(5.69-17.90)
Alcohol Consumption (%)	51.38(42.03-60.73)	37.57(29.15-45.99)	13.81(1.33-26.29)
Insufficient Physical Activity (%)	-	-	
Sedentary Behavior (%)	-	-	

AHA, American Heart Association; BMI, body mass index; GED, General Educational Development; PHQ, Patient Health Questionnaire; PIR, Poverty Impact Ratio. Values are mean for continuous variables and percentage for categorical variables, age-standardized by the age composition ratio of adults with diabetes. Values between brackets indicate 95% CI for constituent ratio. Values between brackets indicate standard deviation (SD) for mean.

Table S5. Age-standardized proportion of unhealthy lifestyle behaviors among adults with diabetes in 2005-2006, by gender

	Men (N =316)	Women (N =306)	Men vs Women
Age (%)			*P*=0.309
20-39 y	7.80(4.15-11.44)	5.47(3.19-7.76)	
40-59 y	35.28(28.86-41.70)	28.65(23.21-34.10)	
≥60 y	56.92(48.25-65.60)	65.87(60.00-71.75)	
Race/Ethnicity (%)			*P*=0.270
Non-Hispanic White	66.85(58.18-75.53)	63.66(55.95-71.36)	
Non-Hispanic Black	17.44(12.29-22.58)	17.22(11.75-22.70)	
Hispanic	12.12(7.37-16.86)	13.24(8.67-17.81)	
Others	3.59(1.06-6.12)	5.88(2.18-9.58)	
Insurance Status (%)			*P*=0.394
Uninsured	9.28(6.60-11.96)	11.10(6.45-15.76)	
Insured	90.72(88.04-93.40)	88.90(84.24-93.55)	
Employment Status (%)			*P*=0.019
Unemployed	46.62(39.45-53.79)	30.01(21.82-38.20)	
Employed	53.38(46.21-60.55)	69.99(61.80-78.18)	
Marital Status (%)			*P*＜0.001
Married or living with partner	76.30(70.28-82.32)	50.95(45.30-56.61)	
Never married	5.42(1.96-8.87)	5.51(2.63-8.40)	
Widowed, divorced, separated	18.29(11.37-25.20)	43.53(38.12-48.95)	
Education Status (%)			*P*=0.431
Below High School	43.78(32.39-55.16)	39.16(29.69-48.62)	
High school graduate or GED	29.91(22.35-37.47)	32.19(26.81-37.56)	
Some college or Above	26.21(20.87-31.56)	28.66(22.05-35.26)	
Family Income Status (%)			*P*=0.048
PIR <1.30	16.45(11.79-21.11)	24.33(17.37-31.30)	
PIR: 1.30-3.49	43.45(31.46-55.43)	46.48(37.04-55.91)	
PIR ≥3.50	36.13(25.36-46.89)	22.78(17.63-27.92)	
AHA Secondary Diet Score	35.69(±1.06)	39.19(±0.98)	-3.51(±1.34)
Poor Diet (%)	37.87(30.17-45.57)	27.19(19.98-34.40)	10.68(0.22-21.15)
PHQ-9 score	2.64(±0.28)	3.82(±0.30)	-1.18(±0.40)
Depression (%)	7.17(2.98-11.37)	8.44(5.71-11.16)	-1.26(-6.23-3.70)
BMI (kg/m^2)	31.81(±0.54)	32.87(±0.51)	-1.06(±0.72)
Obesity (%)	50.47(42.18-58.75)	56.59(50.68-62.50)	-6.12(-16.22-3.97)
Smoking (%)	21.09(15.87-26.32)	14.47(8.54-20.40)	6.63(-1.22-14.47)
Alcohol Consumption (%)	58.42(51.76-65.09)	35.22(30.33-40.11)	23.20(15.00-31.40)
Insufficient Physical Activity (%)	-	-	
Sedentary Behavior (%)	-	-	

AHA, American Heart Association; BMI, body mass index; GED, General Educational Development; PHQ, Patient Health Questionnaire; PIR, Poverty Impact Ratio. Values are mean for continuous variables and percentage for categorical variables, age-standardized by the age composition ratio of adults with diabetes.Values between brackets indicate 95% CI for constituent ratio. Values between brackets indicate standard deviation (SD) for mean.

Table S6. Age-standardized proportion of unhealthy lifestyle behaviors among adults with diabetes in 2007-2008, by gender

	Men (N =509)	Women (N =489)	Men vs Women
Age (%)			*P*=0.362
20-39 y	8.36(6.29-10.43)	7.07(5.26-8.89)	
40-59 y	31.20(25.86-36.54)	33.18(29.58-36.77)	
≥60 y	60.44(55.13-65.75)	59.75(55.63-63.86)	
Race/Ethnicity (%)			*P*=0.047
Non-Hispanic White	70.05(60.19-79.91)	61.32(47.56-75.07)	
Non-Hispanic Black	13.57(8.52-18.63)	20.62(12.92-28.33)	
Hispanic	11.67(7.54-15.80)	12.58(6.80-18.37)	
Others	4.71(0.42-9.00)	5.48(1.83-9.13)	
Insurance Status (%)			*P*=0.479
Uninsured	11.66(8.43-14.88)	10.30(7.03-13.56)	
Insured	88.29(85.12-91.46)	89.70(86.44-92.97)	
Employment Status (%)			*P*=0.096
Unemployed	41.16(33.03-49.28)	32.97(28.32-37.63)	
Employed	58.84(50.72-66.97)	67.03(62.37-71.68)	
Marital Status (%)			*P*＜0.001
Married or living with partner	73.18(69.12-77.24)	48.99(42.97-55.01)	
Never married	10.07(7.13-13.01)	7.51(5.81-9.22)	
Widowed, divorced, separated	16.75(13.42-20.08)	43.50(37.65-49.34)	
Education Status (%)			*P*=0.389
Below High School	42.13(34.73-49.54)	38.66(33.07-44.25)	
High school graduate or GED	28.79(20.26-37.32)	29.17(22.91-35.42)	
Some college or Above	29.08(24.47-33.68)	32.10(27.17-37.02)	
Family Income Status (%)			*P*=0.004
PIR <1.30	18.73(14.92-22.54)	25.64(20.55-30.73)	
PIR: 1.30-3.49	37.29(32.90-41.68)	38.82(34.79-42.85)	
PIR ≥3.50	36.77(31.75-41.80)	23.98(18.62-29.35)	
AHA Secondary Diet Score	35.90(±0.93)	39.02(±0.85)	-3.13(±0.95)
Poor Diet (%)	40.00(34.21-45.78)	30.51(24.14-36.87)	9.49(0.96-18.02)
PHQ-9 score	2.96(±0.13)	4.61(±0.22)	-1.65(±0.22)
Depression (%)	6.79(4.37-9.21)	14.00(9.85-18.15)	-7.21(-11.98--2.44)
BMI (kg/m^2)	31.71(±0.45)	33.49(±0.21)	-1.78(±0.45)
Obesity (%)	53.20(47.54-58.85)	63.74(58.03-69.45)	-10.54(-18.51--2.57)
Smoking (%)	22.66(19.19-26.13)	16.00(12.46-19.54)	6.66(1.75-11.58)
Alcohol Consumption (%)	48.22(44.05-52.39)	31.12(22.72-39.53)	17.10(7.79-26.41)
Insufficient Physical Activity (%)	49.08(41.21-56.94)	67.91(62.54-73.28)	-18.83(-28.28--9.39)
Sedentary Behavior (%)	36.13(29.22-43.05)	35.30(29.07-41.52)	0.84(-8.40-10.07)

AHA, American Heart Association; BMI, body mass index; GED, General Educational Development; PHQ, Patient Health Questionnaire; PIR, Poverty Impact Ratio. Values are mean for continuous variables and percentage for categorical variables, age-standardized by the age composition ratio of adults with diabetes. Values between brackets indicate 95% CI for constituent ratio. Values between brackets indicate standard deviation (SD) for mean.

Table S7. Age-standardized proportion of unhealthy lifestyle behaviors among adults with diabetes in 2009-2010, by gender

	Men (N =516)	Women (N =464)	Men vs Women
Age (%)			*P*=0.361
20-39 y	6.69(4.46-8.93)	4.88(2.46-7.31)	
40-59 y	32.90(25.37-40.44)	25.17(19.01-31.33)	
≥60 y	60.41(52.88-67.94)	69.95(63.83-76.07)	
Race/Ethnicity (%)			*P*=0.008
Non-Hispanic White	65.91(58.99-72.83)	56.95(48.85-65.06)	
Non-Hispanic Black	12.14(7.92-16.36)	18.60(14.17-23.04)	
Hispanic	14.94(7.74-22.14)	15.35(6.19-24.51)	
Others	7.02(3.94-10.09)	9.09(4.96-13.23)	
Insurance Status (%)			*P*=0.217
Uninsured	11.88(8.83-14.93)	10.37(7.06-13.68)	
Insured	88.12(85.07-91.17)	89.63(86.32-92.94)	
Employment Status (%)			*P*=0.003
Unemployed	43.44(37.35-49.52)	28.21(22.84-33.59)	
Employed	56.56(50.48-62.65)	71.79(66.41-77.16)	
Marital Status (%)			*P*＜0.001
Married or living with partner	69.92(65.49-74.35)	51.02(45.01-57.03)	
Never married	8.06(4.60-11.52)	6.99(5.07-8.90)	
Widowed, divorced, separated	21.92(18.34-25.51)	41.20(36.01-46.38)	
Education Status (%)			*P*=0.070
Below High School	53.82(45.51-62.14)	43.48(38.75-48.21)	
High school graduate or GED	20.90(14.62-27.18)	22.18(18.07-26.30)	
Some college or Above	25.27(21.09-29.46)	33.55(28.71-38.40)	
Family Income Status (%)			*P*＜0.001
PIR <1.30	16.00(12.32-19.67)	27.72(22.50-32.94)	
PIR: 1.30-3.49	38.10(32.49-43.72)	38.88(32.64-45.12)	
PIR ≥3.50	38.50(34.72-42.28)	23.44(17.16-29.72)	
AHA Secondary Diet Score	37.07(±0.84)	39.48(±0.57)	-2.41(±0.98)
Poor Diet (%)	34.00(27.89-40.11)	28.99(23.89-34.09)	5.01(-2.88-12.90)
PHQ-9 score	2.73(±0.15)	4.47(±0.38)	-1.74(±0.50)
Depression (%)	6.76(5.17-8.36)	13.00(8.77-17.24)	-6.24(-10.73--1.75)
BMI (kg/m^2)	32.06(±0.49)	34.03(±0.53)	-1.97(±0.72)
Obesity (%)	58.71(51.71-65.72)	66.83(61.23-72.42)	-8.12(-17.01-0.78)
Smoking (%)	22.74(19.81-25.66)	11.78(8.34-15.23)	10.95(6.47-15.43)
Alcohol Consumption (%)	61.69(57.83-65.54)	38.13(32.43-43.82)	23.56(16.73-30.38)
Insufficient Physical Activity (%)	50.85(45.29-56.42)	67.60(63.40-71.81)	-16.75(-23.67--9.83)
Sedentary Behavior (%)	40.39(34.31-46.47)	33.44(27.25-39.63)	6.95(-1.66-15.56)

AHA, American Heart Association; BMI, body mass index; GED, General Educational Development; PHQ, Patient Health Questionnaire; PIR, Poverty Impact Ratio. Values are mean for continuous variables and percentage for categorical variables, age-standardized by the age composition ratio of adults with diabetes. Values between brackets indicate 95% CI for constituent ratio. Values between brackets indicate standard deviation (SD) for mean.

Table S8. Age-standardized proportion of unhealthy lifestyle behaviors among adults with diabetes in 2011-12, by gender

	Men (N =470)	Women (N =441)	Men vs Women
Age (%)			P=0.718
20-39 y	6.33(3.46-9.21)	6.99(4.89-9.10)	
40-59 y	35.43(27.26-43.60)	31.56(26.38-36.74)	
≥60 y	58.24(49.46-67.01)	61.45(56.46-66.44)	
Race/Ethnicity (%)			P=0.314
Non-Hispanic White	59.88(49.05-70.72)	54.46(43.03-65.88)	
Non-Hispanic Black	15.11(8.48-21.75)	18.97(9.65-28.30)	
Hispanic	14.73(7.90-21.57)	16.26(8.60-23.91)	
Others	10.27(6.16-14.38)	10.31(4.83-15.79)	
Insurance Status (%)			P=0.647
Uninsured	15.89(12.10-19.68)	14.31(10.22-18.40)	
Insured	84.11(80.32-87.90)	85.30(80.98-89.63)	
Employment Status (%)			P<0.001
Unemployed	47.10(39.43-54.77)	33.37(25.22-41.51)	
Employed	52.90(45.23-60.57)	66.63(58.49-74.78)	
Marital Status (%)			P<0.001
Married or living with partner	68.72(61.65-75.78)	49.07(43.93-54.22)	
Never married	10.55(6.30-14.80)	10.35(7.54-13.15)	
Widowed, divorced, separated	20.64(16.06-25.21)	40.34(35.28-45.40)	
Education Status (%)			P=0.016
Below High School	52.34(45.75-58.93)	42.09(34.03-50.14)	
High school graduate or GED	21.77(15.09-28.44)	27.96(22.00-33.92)	
Some college or Above	25.81(19.14-32.48)	29.95(22.36-37.55)	
Family Income Status (%)			P=0.005
PIR <1.30	23.95(17.68-30.23)	35.92(26.71-45.12)	
PIR: 1.30-3.49	34.14(26.76-41.52)	37.08(28.63-45.52)	
PIR ≥3.50	34.84(26.10-43.58)	19.59(11.59-27.59)	
AHA Secondary Diet Score	37.20(±0.91)	39.30(±0.83)	-2.11(±1.13)
Poor Diet (%)	32.28(26.30-38.26)	31.85(24.69-39.01)	0.43(-8.83-9.69)
PHQ-9 score	2.96(±0.31)	4.78(±0.43)	-1.82(±0.53)
Depression (%)	5.78(3.11-8.46)	15.92(10.71-21.14)	-10.14(-15.95--4.33)
BMI (kg/m^2)	31.95(±0.92)	33.57(±0.56)	-1.63(±1.45)
Obesity (%)	55.02(47.04-63.01)	61.96(53.48-70.44)	-6.94(-18.49-4.62)
Smoking (%)	21.99(14.99-28.98)	14.38(8.87-19.88)	7.61(-1.22-16.44)
Alcohol Consumption (%)	61.51(54.64-68.37)	44.24(35.95-52.53)	17.27(6.59-27.95)
Insufficient Physical Activity (%)	51.36(45.43-57.29)	61.24(52.35-70.12)	-9.87(-20.47-0.73)
Sedentary Behavior (%)	53.33(47.13-59.54)	42.66(39.09-46.22)	10.68(3.58-17.78)

AHA, American Heart Association; BMI, body mass index; GED, General Educational Development; PHQ, Patient Health Questionnaire; PIR, Poverty Impact Ratio. Values are mean for continuous variables and percentage for categorical variables, age-standardized by the age composition ratio of adults with diabetes. Values between brackets indicate 95% CI for constituent ratio. Values between brackets indicate standard deviation (SD) for mean.

Table S9. Age-standardized proportion of unhealthy lifestyle behaviors among adults with diabetes in 2013-14, by gender

	Men (N =473)	Women (N =446)	Men vs Women
Age (%)			*P*=0.274
20-39 y	4.41(2.36-6.46)	6.61(4.25-8.97)	
40-59 y	32.87(26.33-39.40)	29.26(24.63-33.88)	
≥60 y	62.73(55.72-69.74)	64.13(59.75-68.51)	
Race/Ethnicity (%)			*P*=0.108
Non-Hispanic White	65.64(56.98-74.30)	60.94(54.01-67.87)	
Non-Hispanic Black	11.99(8.11-15.86)	16.61(11.08-22.14)	
Hispanic	13.92(7.68-20.16)	14.72(9.29-20.14)	
Others	8.45(5.30-11.60)	7.74(4.94-10.53)	
Insurance Status (%)			*P*=0.094
Uninsured	11.08(7.50-14.66)	8.18(5.82-10.54)	
Insured	88.92(85.34-92.50)	91.82(89.46-94.18)	
Employment Status (%)			*P*=0.002
Unemployed	44.34(38.66-50.02)	30.60(25.01-36.18)	
Employed	55.66(49.98-61.34)	69.40(63.82-74.99)	
Marital Status (%)			*P*＜0.001
Married or living with partner	72.08(68.17-75.98)	50.73(45.61-55.85)	
Never married	7.77(5.70-9.83)	9.16(6.51-11.80)	
Widowed, divorced, separated	20.16(16.94-23.37)	40.11(34.97-45.25)	
Education Status (%)			*P*=0.235
Below High School	56.02(47.72-64.32)	49.44(42.79-56.08)	
High school graduate or GED	22.48(17.19-27.77)	26.69(21.81-31.57)	
Some college or Above	21.36(14.96-27.76)	23.87(18.44-29.30)	
Family Income Status (%)			*P*=0.027
PIR <1.30	23.95(19.61-28.29)	30.92(25.37-36.47)	
PIR: 1.30-3.49	32.57(26.81-38.34)	37.67(31.66-43.67)	
PIR ≥3.50	36.96(30.50-43.42)	26.05(17.96-34.14)	
AHA Secondary Diet Score	38.02(±0.95)	40.66(±1.01)	-2.65(±1.31)
Poor Diet (%)	36.61(31.36-41.85)	26.03(21.52-30.53)	10.58(3.72-17.43)
PHQ-9 score	3.05(±0.18)	4.99(±0.31)	-1.94(±0.30)
Depression (%)	7.36(4.15-10.56)	16.99(12.02-21.97)	-9.64(-15.51--3.77)
BMI (kg/m^2)	32.25(±0.57)	33.58(±0.51)	-1.33(±0.57)
Obesity (%)	55.45(47.41-63.49)	63.48(57.82-69.15)	-8.03(-17.79-1.72)
Smoking (%)	17.34(13.84-20.85)	14.65(11.23-18.07)	2.69(-2.17-7.55)
Alcohol Consumption (%)	58.37(52.47-64.26)	51.93(45.49-58.37)	6.44(-2.22-15.10)
Insufficient Physical Activity (%)	52.53(45.67-59.39)	59.39(55.16-63.62)	-6.86(-14.86-1.14)
Sedentary Behavior (%)	62.01(54.31-69.72)	58.97(52.01-65.92)	3.05(-7.25-13.34)

AHA, American Heart Association; BMI, body mass index; GED, General Educational Development; PHQ, Patient Health Questionnaire; PIR, Poverty Impact Ratio. Values are mean for continuous variables and percentage for categorical variables, age-standardized by the age composition ratio of adults with diabetes. Values between brackets indicate 95% CI for constituent ratio. Values between brackets indicate standard deviation (SD) for mean.

Table S10. Age-standardized proportion of unhealthy lifestyle behaviors among adults with diabetes in 2015-16, by gender

	Men (N =558)	Women (N =481)	Men vs Women
Age (%)			*P*=0.983
20-39 y	7.68(5.80-9.56)	7.71(5.43-9.99)	
40-59 y	30.94(26.49-35.40)	29.44(22.40-36.47)	
≥60 y	61.38(56.48-66.28)	62.86(56.21-69.51)	
Race/Ethnicity (%)			*P*=0.061
Non-Hispanic White	62.62(54.68-70.56)	54.85(42.69-67.01)	
Non-Hispanic Black	11.37(7.12-15.62)	16.64(9.09-24.18)	
Hispanic	15.51(8.55-22.46)	19.09(10.06-28.12)	
Others	10.50(6.34-14.66)	9.43(6.46-12.40)	
Insurance Status (%)			*P*=0.946
Uninsured	7.69(5.19-10.20)	7.83(5.11-10.56)	
Insured	92.25(89.75-94.74)	92.17(89.44-94.89)	
Employment Status (%)			*P*=0.189
Unemployed	46.96(40.66-53.27)	39.65(34.01-45.29)	
Employed	53.04(46.73-59.34)	60.12(54.46-65.78)	
Marital Status (%)			*P*=0.002
Married or living with partner	70.56(65.78-75.34)	53.76(47.47-60.05)	
Never married	10.25(6.48-14.03)	10.51(5.64-15.37)	
Widowed, divorced, separated	19.19(14.52-23.86)	35.73(28.97-42.50)	
Education Status (%)			*P*=0.310
Below High School	58.47(53.00-63.95)	54.06(48.38-59.73)	
High school graduate or GED	22.27(18.75-25.78)	21.69(16.57-26.81)	
Some college or Above	19.17(14.77-23.58)	24.16(19.17-29.16)	
Family Income Status (%)			*P*=0.004
PIR <1.30	19.88(16.21-23.55)	30.35(23.39-37.31)	
PIR: 1.30-3.49	32.91(25.61-40.21)	35.30(29.75-40.85)	
PIR ≥3.50	37.16(29.42-44.91)	24.19(18.08-30.30)	
AHA Secondary Diet Score	37.43(±0.80)	38.53(±0.83)	-1.10(±1.07)
Poor Diet (%)	30.14(23.66-36.62)	31.67(24.53-38.82)	-1.53(-11.10-8.04)
PHQ-9 score	2.73(±0.25)	5.10(±0.32)	-2.37(±0.45)
Depression (%)	5.18(3.47-6.89)	16.35(12.02-20.69)	-11.17(-15.80--6.55)
BMI (kg/m^2)	31.49(±0.38)	33.90(±0.36)	-2.41(±0.59)
Obesity (%)	53.62(46.63-60.60)	69.11(64.54-73.68)	-15.49(-23.78--7.21)
Smoking (%)	18.30(14.38-22.22)	13.12(8.73-17.50)	5.18(-0.65-11.02)
Alcohol Consumption (%)	64.01(56.25-71.77)	46.39(38.91-53.87)	17.62(6.93-28.31)
Insufficient Physical Activity (%)	39.50(34.68-44.31)	58.21(52.10-64.32)	-18.71(-26.43--10.99)
Sedentary Behavior (%)	45.55(37.26-53.85)	42.76(35.19-50.33)	2.79(-8.35-13.94)

AHA, American Heart Association; BMI, body mass index; GED, General Educational Development; PHQ, Patient Health Questionnaire; PIR, Poverty Impact Ratio. Values are mean for continuous variables and percentage for categorical variables, age-standardized by the age composition ratio of adults with diabetes. Values between brackets indicate 95% CI for constituent ratio. Values between brackets indicate standard deviation (SD) for mean.

Table S11. Gender differences in temporal trends for unhealthy lifestyle behaviors among adults with diabetes between 1999 and 2018 by age, race, marital status, and insurance status

		By Age				*By Race/Ethnicity*					*By Marital Status*				*By Insurance Status*		
	Overall	20-39	40-59	≥60	*P*	NH-W	NH-B	H	Others	*P*	Marry	Never	Widow	*P*	Uninsured	Insured	*P*
AHA Secondary Diet Score	0.245	0.492	0.129	0.000	0.989	0.000	0.005	0.000	0.111	0.928	0.000	0.386	0.000	0.106	0.016	0.000	0.571
Poor Diet	0.037	0.656	0.552	0.000	0.342	0.011	0.046	0.010	0.454	0.785	0.001	0.818	0.009	0.023	0.039	0.001	0.415
PHQ-9 Score	0.425	0.002	0.000	0.000	0.027	0.000	0.000	0.000	0.004	0.597	0.000	0.001	0.001	0.409	0.009	0.000	0.001
Depression	0.841	0.004	0.000	0.000	0.108	0.000	0.018	0.000	0.310	0.330	0.000	0.010	0.191	0.640	0.131	0.000	0.654
BMI	0.019	0.000	0.000	0.000	0.356	0.000	0.000	0.000	0.550	0.009	0.000	0.000	0.002	0.279	0.001	0.004	0.243
Obesity	0.078	0.065	0.000	0.000	0.991	0.000	0.000	0.000	0.705	0.061	0.000	0.000	0.037	0.351	0.017	0.000	0.140
Smoking	0.073	0.060	0.010	0.000	0.300	0.004	0.000	0.000	0.000	0.513	0.002	0.063	0.000	0.521	0.000	0.000	0.879
Alcohol Consumption	0.170	0.080	0.000	0.000	0.326	0.000	0.000	0.000	0.000	0.623	0.000	0.105	0.000	0.339	0.000	0.000	0.419
Insufficient Physical Activity	0.372	0.001	0.000	0.000	0.204	0.000	0.000	0.000	0.052	0.089	0.000	0.149	0.000	0.259	0.000	0.000	0.000
Sedentary Behavior	0.827	0.443	0.257	0.020	0.008	0.260	0.173	0.808	0.001	0.459	0.000	0.015	0.154	0.680	0.643	0.013	0.000

BMI, body mass index; NH-W: non-Hispanic White; NH-B, non-Hispanic Black; H, Hispanic. P-values are derived from regression models including an interaction term between gender and calendar period modelled as continuous variable. P-values for interaction indicate whether gender differences in trends differed by age, Race/ethnicity, marital status and insurance status.

Table S12. Gender differences in temporal trends for unhealthy lifestyle behaviors among adults with diabetes between 1999 and 2018 by employment status, education, and family income

		By Employment Status			*By Education Status*				*By Family Income Status*			
	Overall	Employed	Unemployed	*P*	College	High	Below	*P*	PIR≤1.3	1.3<PIR<3.5	PIR≥3.5	*P*
AHA Secondary Diet Score	0.245	0.021	0.000	0.094	0.033	0.001	0.000	0.643	0.000	0.000	0.004	0.716
Poor Diet	0.037	0.575	0.000	0.005	0.390	0.204	0.000	0.933	0.005	0.001	0.096	0.670
PHQ-9 Score	0.425	0.000	0.000	0.263	0.000	0.000	0.000	0.054	0.000	0.000	0.000	0.115
Depression	0.841	0.006	0.000	0.645	0.000	0.001	0.001	0.124	0.000	0.007	0.148	0.422
BMI	0.019	0.000	0.000	0.510	0.000	0.000	0.000	0.981	0.000	0.000	0.002	0.279
Obesity	0.078	0.000	0.000	0.000	0.000	0.000	0.003	0.119	0.001	0.004	0.003	0.472
Smoking	0.073	0.002	0.000	0.003	0.081	0.000	0.000	0.682	0.000	0.000	0.063	0.373
Alcohol Consumption	0.170	0.027	0.000	0.000	0.000	0.000	0.000	0.915	0.000	0.000	0.015	0.832
Insufficient Physical Activity	0.372	0.000	0.000	0.596	0.000	0.005	0.000	0.834	0.000	0.001	0.002	0.161
Sedentary Behavior	0.827	0.144	0.042	0.952	0.220	0.248	0.093	0.728	0.578	0.698	0.128	0.377

BMI, body mass index. P-values are derived from regression models including an interaction term between gender and calendar period modelled as continuous variable. P-values for interaction indicate whether gender differences in trends differed by employment status, education status and family Income Status.

College: Some college or Above; High: High school graduate or General Educational Development; Below: Below High School; PIR: Poverty Impact Ratio.

Figure Legends

Figure S1. Gender differences in unhealthy lifestyle behaviors among adults with diabetes by age . Odds ratios with 95% confidence interval (OR, 95% CI) of women vs men. Women served as the reference group. ** P≤0.001, * P<0.05.

Figure S2. Gender differences in unhealthy lifestyle behaviors among adults with diabetes by race/ethnicity. Odds ratios with 95% confidence interval (OR, 95% CI) of women vs men. Women served as the reference group. ** P≤0.001, * P<0.05.

Figure S3. Gender differences in unhealthy lifestyle behaviors among adults with diabetes by marital status. Odds ratios with 95% confidence interval (OR, 95% CI) of women vs men. Women served as the reference group. ** P≤0.001, * P<0.05.

Figure S4. Gender differences in unhealthy lifestyle behaviors among adults with diabetes by insurance status. Odds ratios with 95% confidence interval (OR, 95% CI) of women vs men. Women served as the reference group. ** P≤0.001, * P<0.05.

Figure S5. Gender differences in unhealthy lifestyle behaviors among adults with diabetes by employment status. Odds ratios with 95% confidence interval (OR, 95% CI) of women vs men. Women served as the reference group. ** P≤0.001, * P<0.05.

Figure S6. Gender differences in unhealthy lifestyle behaviors among adults

with diabetes by education. Odds ratios with 95% confidence interval (OR, 95% CI) of women vs men. Women served as the reference group. ** P≤0.001, * P<0.05.

Figure S7. Gender differences in unhealthy lifestyle behaviors among adults with diabetes by family income. Odds ratios with 95% confidence interval (OR, 95% CI) of women vs men. Women served as the reference group. ** P≤0.001, * P<0.05.

Figure S1

Unhealthy Lifestyles Among Diabetics	Age (Y)	Women	Men	OR
Poor Diet (%)	20 ~ 39	47.52 (38.58-56.47)	44.50 (35.61-53.38)	0.89 (0.53-1.49)
	40 ~ 59	35.25 (30.80-39.69)	36.93 (32.73-41.14)	1.08 (0.85-1.37)
	≥ 60	25.43 (23.06-27.80)	33.06 (30.52-35.59)	1.45 (1.22-1.71)**
Depression (%)	20 ~ 39	13.54 (8.74-18.34)	5.97 (3.11-8.84)	0.37 (0.19-0.72)*
	40 ~ 59	12.37 (10.01-14.73)	6.95 (5.21-8.69)	0.52 (0.37-0.74)**
	≥ 60	9.71 (7.92-11.51)	4.90 (3.78-6.01)	0.44 (0.32-0.61)**
Obesity (%)	20 ~ 39	69.71 (63.04-76.38)	59.96 (52.48-67.44)	0.66 (0.42-1.02)
	40 ~ 59	72.00 (68.86-75.13)	59.23 (55.48-62.98)	0.55 (0.44-0.68)**
	≥ 60	56.81 (54.11-59.52)	49.40 (46.65-52.16)	0.74 (0.63-0.87)**
Smoking (%)	20 ~ 39	29.13 (22.64-35.62)	38.06 (31.07-45.04)	1.51 (0.99-2.30)
	40 ~ 59	22.35 (19.29-25.42)	28.39 (25.18-31.60)	1.38 (1.08-1.75)*
	≥ 60	8.92 (7.42-10.42)	14.96 (12.96-16.97)	1.80 (1.42-2.27)**
Alcohol Consumption (%)	20 ~ 39	62.58 (55.08-70.08)	69.45 (62.44-76.46)	1.44 (0.96-2.15)
	40 ~ 59	50.64 (46.27-55.01)	65.13 (61.53-68.73)	1.95 (1.53-2.48)**
	≥ 60	35.98 (32.95-39.01)	53.73 (50.64-56.82)	2.02 (1.70-2.40)**
Insufficient Physical Activity (%)	20 ~ 39	30.02 (24.32-35.73)	18.58 (13.45-23.70)	0.47 (0.31-0.73)**
	40 ~ 59	38.82 (35.07-42.56)	28.49 (25.56-31.41)	0.54 (0.43-0.68)**
	≥ 60	45.03 (42.27-47.80)	37.58 (34.57-40.60)	0.58 (0.48-0.70)**
Sedentary Behavior (%)	20 ~ 39	24.46 (18.82-30.11)	21.71 (15.91-27.51)	0.83 (0.51-1.34)
	40 ~ 59	28.21 (24.66-31.76)	30.93 (27.35-34.50)	1.17 (0.89-1.53)
	≥ 60	29.28 (26.49-32.07)	35.12 (31.86-38.38)	1.26 (1.04-1.54)*

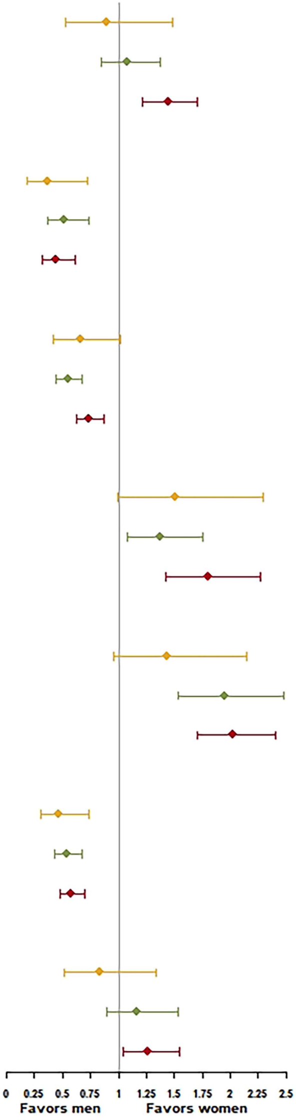

Figure S2

Unhealthy Lifestyles Among Diabetics	Race/Ethnicity	Women	Men	OR
Poor Diet (%)	Non-Hispanic White	29.81 (26.59-33.02)	35.26 (32.30-38.21)	1.28 (1.06-1.55)**
	Non-Hispanic Black	37.95 (34.02-41.89)	43.01 (39.71-46.32)	1.24 (1.01-1.52)*
	Hispanic	25.98 (21.82-30.13)	32.69 (29.17-36.22)	1.38 (1.08-1.77)**
	Others	21.38 (14.99-27.77)	24.70 (17.34-32.07)	1.21 (0.74-1.97)
Depression (%)	Non-Hispanic White	11.00 (8.77-13.22)	5.14 (3.96-6.32)	0.42 (0.30-0.58)**
	Non-Hispanic Black	9.65 (7.60-11.70)	6.65 (5.01-8.29)	0.62 (0.42-0.91)*
	Hispanic	13.27 (10.90-15.64)	7.34 (5.41-9.27)	0.50 (0.35-0.69)**
	Others	7.06 (3.52-10.60)	5.25 (2.55-7.94)	0.67 (0.23-1.05)
Obesity (%)	Non-Hispanic White	64.48 (61.17-67.79)	56.66 (53.70-59.62)	0.72 (0.60-0.86)**
	Non-Hispanic Black	68.47 (65.63-71.32)	52.99 (49.91-56.08)	0.52 (0.44-0.62)**
	Hispanic	60.65 (56.71-64.58)	49.19 (45.69-52.68)	0.60 (0.49-0.72)**
	Others	35.61 (29.61-41.60)	33.49 (25.54-41.44)	0.92 (0.62-1.38)
Smoking (%)	Non-Hispanic White	14.06 (11.76-16.37)	18.72 (16.69-20.76)	1.41 (1.12-1.77)*
	Non-Hispanic Black	18.37 (15.95-20.78)	27.46 (24.74-30.18)	1.69 (1.37-2.08)**
	Hispanic	12.39 (10.18-14.61)	21.76 (18.98-24.54)	1.97 (1.52-2.56)**
	Others	10.78 (6.26-15.30)	26.58 (19.26-33.89)	3.00 (1.79-5.00)**
Alcohol Consumption (%)	Non-Hispanic White	46.85 (42.76-50.93)	59.99 (56.47-63.51)	1.68 (1.36-2.06)**
	Non-Hispanic Black	38.65 (35.34-41.95)	52.68 (49.02-56.34)	1.78 (1.48-2.13)**
	Hispanic	35.98 (32.20-39.75)	60.19 (56.60-63.79)	2.86 (2.31-3.54)**
	Others	27.94 (22.29-33.59)	52.18 (44.93-59.43)	3.27 (2.11-5.08)**
Insufficient Physical Activity (%)	Non-Hispanic White	40.53 (37.17-43.88)	33.09 (29.74-36.44)	0.61 (0.49-0.76)**
	Non-Hispanic Black	45.33 (41.04-49.62)	34.38 (31.07-37.70)	0.48 (0.39-0.60)**
	Hispanic	45.20 (39.74-50.66)	31.60 (27.42-35.78)	0.46 (0.37-0.58)**
	Others	41.25 (34.10-48.40)	37.11 (30.57-43.65)	0.65 (0.42-1.00)
Sedentary Behavior (%)	Non-Hispanic White	32.08 (28.85-35.31)	35.34 (31.71-38.98)	1.13 (0.91-1.40)
	Non-Hispanic Black	27.52 (24.25-30.79)	30.73 (27.35-34.10)	1.15 (0.94-1.41)
	Hispanic	20.28 (16.92-23.64)	20.12 (16.39-23.85)	0.97 (0.76-1.23)
	Others	21.61 (16.66-26.55)	37.62 (31.19-44.05)	2.06 (1.40-3.04)**

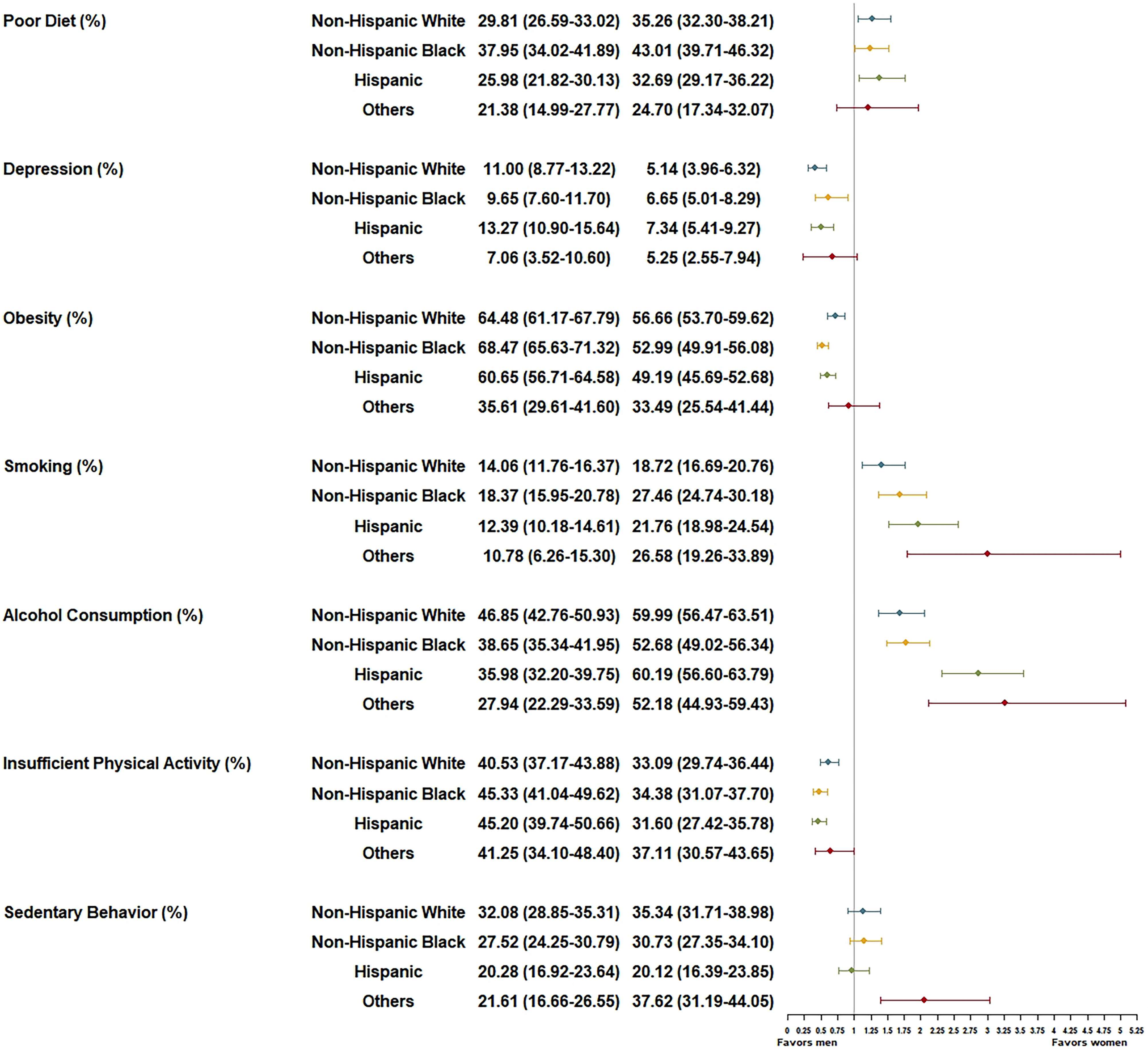

Figure S3

Unhealthy Lifestyles Among Diabetics	Marital Status	Women	Men	OR
Poor Diet (%)	Married or Living with Partner	27.86 (24.76-30.96)	33.82 (31.38-36.25)	1.32 (1.13-1.56)**
	Never Married	38.62 (30.95-46.29)	39.86 (32.49-47.23)	1.05 (0.69-1.61)
	Widowed, Divorced, Separated	30.85 (27.91-33.80)	37.71 (32.85-42.57)	1.36 (1.08-1.70)*
Depression (%)	Married or Living with Partner	10.05 (8.04-12.06)	4.44 (3.55-5.32)	0.41 (0.32-0.53)**
	Never Married	14.64 (9.62-19.66)	7.59 (4.66-10.51)	0.45 (0.24-0.81)*
	Widowed, Divorced, Separated	11.03 (9.33-12.73)	9.59 (6.82-12.36)	0.77 (0.51-1.14)
Obesity (%)	Married or Living with Partner	62.77 (59.52-66.02)	54.38 (51.76-57.01)	0.70 (0.60-0.83)**
	Never Married	76.45 (71.30-81.60)	48.63 (41.98-55.28)	0.28 (0.19-0.42)**
	Widowed, Divorced, Separated	58.26 (55.09-61.42)	51.46 (46.76-56.17)	0.77 (0.60-0.98)*
Smoking (%)	Married or Living with Partner	13.12 (10.97-15.27)	18.08 (16.14-20.02)	1.46 (1.16-1.85)*
	Never Married	21.13 (16.32-25.93)	29.38 (22.55-36.20)	1.56 (0.98-2.47)
	Widowed, Divorced, Separated	14.58 (12.48-16.68)	27.40 (22.88-31.91)	2.21 (1.65-2.97)**
Alcohol Consumption (%)	Married or Living with Partner	43.89 (40.28-47.50)	59.42 (56.38-62.46)	1.87 (1.55-2.26)**
	Never Married	50.51 (44.08-56.94)	56.81 (49.48-64.13)	1.44 (0.93-2.23)
	Widowed, Divorced, Separated	38.48 (34.89-42.07)	56.52 (52.34-60.70)	2.09 (1.63-2.68)**
Insufficient Physical Activity (%)	Married or Living with Partner	41.17 (37.78-44.57)	32.84 (30.22-35.46)	0.64 (0.52-0.77)**
	Never Married	38.29 (31.80-44.77)	35.80 (28.51-43.09)	0.71 (0.44-1.13)
	Widowed, Divorced, Separated	44.91 (41.73-48.09)	35.68 (31.39-39.98)	0.47 (0.37-0.61)**
Sedentary Behavior (%)	Married or Living with Partner	26.83 (23.91-29.74)	32.83 (29.69-35.97)	1.44 (1.20-1.73)**
	Never Married	34.16 (27.86-40.47)	27.17 (21.03-33.31)	0.57 (0.36-0.89)*
	Widowed, Divorced, Separated	30.24 (27.37-33.12)	36.86 (32.22-41.50)	1.20 (0.94-1.54)

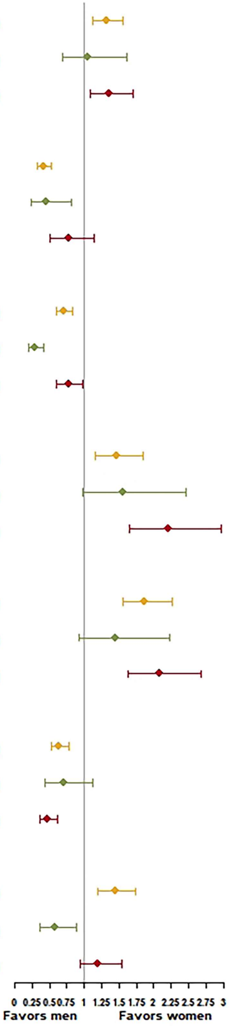

Figure S4

Unhealthy Lifestyles Among Diabetics	Insurance Status	Women	Men	OR
Poor Diet (%)	Uninsured	31.88 (26.05-37.71)	40.66 (34.71-46.60)	1.46 (1.02-2.09)*
	Insured	29.68 (27.38-31.99)	34.44 (32.19-36.68)	1.24 (1.09-1.42)**
Depression (%)	Uninsured	12.52 (8.89-16.15)	9.20 (5.39-13.00)	0.65 (0.38-1.13)
	Insured	10.62 (9.15-12.08)	5.25 (4.46-6.04)	0.44 (0.36-0.55)**
Obesity (%)	Uninsured	64.30 (58.59-70.02)	54.88 (49.50-60.26)	0.67 (0.49-0.93)*
	Insured	61.95 (59.77-64.12)	53.17 (50.62-55.72)	0.69 (0.60-0.79)**
Smoking (%)	Uninsured	24.94 (20.08-29.80)	39.56 (34.09-45.04)	1.98 (1.39-2.82)**
	Insured	13.13 (11.67-14.59)	18.72 (17.17-20.26)	1.52 (1.30-1.79)**
Alcohol Consumption (%)	Uninsured	42.37 (36.90-47.84)	64.91 (59.74-70.08)	2.72 (1.91-3.85)**
	Insured	42.24 (39.51-44.97)	57.64 (54.95-60.33)	1.86 (1.60-2.17)**
Insufficient Physical Activity (%)	Uninsured	35.80 (30.68-40.91)	25.56 (20.71-30.41)	0.44 (0.32-0.62)**
	Insured	42.94 (40.44-45.44)	34.40 (31.95-36.85)	0.58 (0.50-0.68)**
Sedentary Behavior (%)	Uninsured	19.37 (14.56-24.19)	23.34 (18.15-28.52)	1.11 (0.71-1.72)
	Insured	29.73 (27.52-31.94)	34.11 (31.43-36.79)	1.23 (1.05-1.44)*

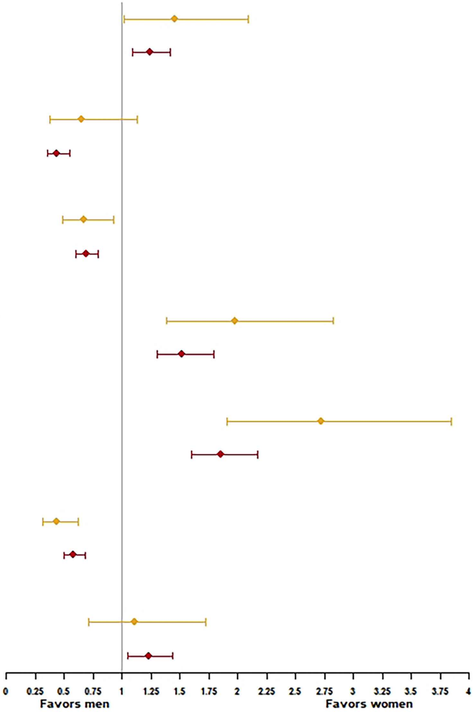

Figure S5

Unhealthy Lifestyles Among Diabetics	Employment Status	Women	Men	OR
Poor Diet (%)	Employed	34.36 (29.95-38.76)	35.89 (32.34-39.43)	1.07 (0.85-1.35)
	Unemployed	27.97 (25.56-30.38)	34.46 (31.99-36.94)	1.35 (1.17-1.56)**
Depression (%)	Employed	7.25 (5.01-9.50)	3.54 (2.24-4.83)	0.48 (0.29-0.80)*
	Unemployed	12.42 (10.84-14.00)	7.34 (6.16-8.51)	0.51 (0.40-0.64)**
Obesity (%)	Employed	70.43 (67.25-73.62)	57.29 (53.59-60.99)	0.55 (0.45-0.68)**
	Unemployed	58.31 (55.82-60.80)	50.08 (47.57-52.60)	0.73 (0.63-0.84)**
Smoking (%)	Employed	16.59 (13.78-19.40)	22.93 (20.33-25.52)	1.50 (1.17-1.92)*
	Unemployed	13.21 (11.52-14.90)	19.18 (17.15-21.21)	1.56 (1.28-1.90)**
Alcohol Consumption (%)	Employed	60.86 (56.71-65.01)	67.04 (63.89-70.19)	1.31 (1.03-1.66)*
	Unemployed	33.42 (30.56-36.28)	51.56 (48.55-54.58)	2.11 (1.79-2.50)**
Insufficient Physical Activity (%)	Employed	37.36 (32.81-41.91)	25.25 (21.99-28.51)	0.53 (0.41-0.68)**
	Unemployed	44.41 (41.68-47.13)	39.94 (36.87-43.01)	0.65 (0.54-0.80)**
Sedentary Behavior (%)	Employed	29.56 (26.03-33.09)	32.01 (28.45-35.58)	1.21 (0.94-1.55)
	Unemployed	28.20 (25.84-30.57)	33.58 (30.23-36.93)	1.21 (1.01-1.46)*

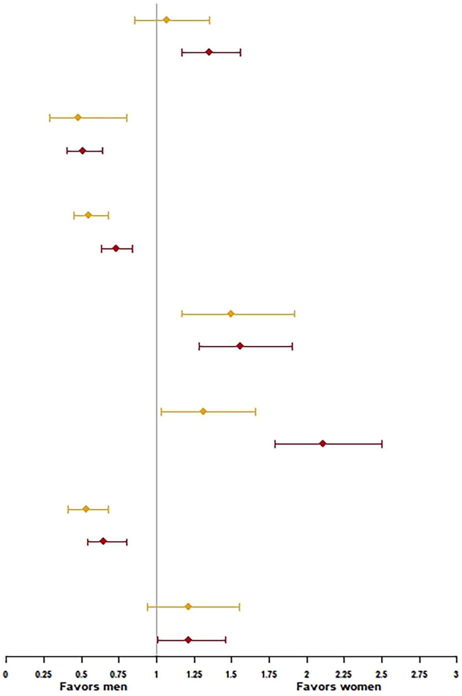

Figure S6

Unhealthy Lifestyles Among Diabetics	Education Status	Women	Men	OR
Poor Diet (%)	Some College	29.33 (25.96-32.69)	31.16 (27.94-34.39)	1.09 (0.90-1.33)
	High school graduated or GED	30.69 (26.19-35.19)	34.86 (30.49-39.22)	1.21 (0.90-1.62)
	Below High school	30.13 (26.47-33.79)	43.29 (39.44-47.14)	1.77 (1.44-2.17)**
Depression (%)	Some College	9.29 (7.39-11.19)	4.23 (2.82-5.63)	0.42 (0.28-0.64)**
	High school graduated or GED	11.37 (8.48-14.25)	5.82 (3.92-7.72)	0.45 (0.29-0.70)**
	Below High school	12.37 (10.50-14.24)	8.28 (6.39-10.17)	0.60 (0.45-0.80)**
Obesity (%)	Some College	64.96 (61.62-68.30)	55.99 (52.48-59.49)	0.68 (0.56-0.82)**
	High school graduated or GED	64.96 (61.12-68.80)	53.41 (48.89-57.93)	0.63 (0.49-0.80)**
	Below High school	55.56 (52.26-58.86)	47.84 (44.31-51.36)	0.72 (0.59-0.89)*
Smoking (%)	Some College	13.11 (10.71-15.51)	16.29 (13.96-18.61)	1.29 (0.97-1.71)
	High school graduated or GED	15.39 (12.33-18.45)	23.92 (20.26-27.58)	1.73 (1.30-2.30)**
	Below High school	15.08 (12.66-17.50)	27.44 (24.78-30.10)	2.13 (1.72-2.65)**
Alcohol Consumption (%)	Some College	54.03 (50.51-57.56)	67.61 (64.24-70.98)	1.77 (1.43-2.20)**
	High school graduated or GED	41.49 (37.12-45.87)	54.35 (49.51-59.18)	1.70 (1.32-2.20)**
	Below High school	25.55 (22.49-28.61)	43.71 (40.15-47.27)	2.28 (1.86-2.79)**
Insufficient Physical Activity (%)	Some College	43.29 (39.59-46.98)	31.38 (28.34-34.41)	0.52 (0.42-0.65)**
	High school graduated or GED	37.88 (34.50-41.27)	33.82 (29.67-37.98)	0.71 (0.56-0.90)*
	Below High school	44.36 (40.65-48.06)	37.29 (33.69-40.89)	0.58 (0.45-0.73)**
Sedentary Behavior (%)	Some College	35.22 (31.75-38.69)	38.25 (34.95-41.55)	1.14 (0.93-1.40)
	High school graduated or GED	25.21 (22.11-28.31)	29.51 (24.89-34.12)	1.22 (0.87-1.69)
	Below High school	22.04 (19.07-25.02)	25.06 (21.77-28.35)	1.18 (0.97-1.44)

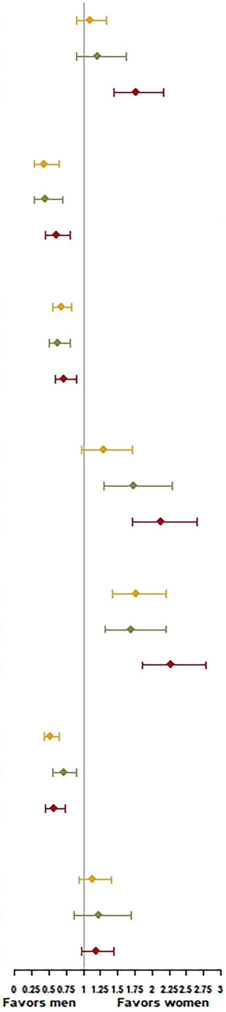

Figure S7

Unhealthy Lifestyles Among Diabetics	Family Income Levels	Women	Men	OR
Poor Diet (%)	PIR ≤ 1.30	37.12 (33.17-41.07)	44.59 (40.61-48.58)	1.37 (1.10-1.69)*
	1.3 < PIR < 3.5	27.90 (24.89-30.91)	35.92 (32.07-39.76)	1.45 (1.17-1.79)**
	PIR ≥ 3.5	25.03 (20.64-29.42)	30.45 (25.99-34.90)	1.31 (0.96-1.80)
Depression (%)	PIR ≤ 1.30	17.98 (15.51-20.44)	9.51 (7.33-11.69)	0.45 (0.34-0.60)**
	1.3 < PIR < 3.5	9.35 (7.13-11.56)	5.81 (4.46-7.16)	0.57 (0.38-0.85)*
	PIR ≥ 3.5	5.56 (3.31-7.81)	3.54 (2.06-5.01)	0.62 (0.33-1.18)
Obesity (%)	PIR ≤ 1.30	59.07 (55.58-62.55)	49.74 (45.95-53.52)	0.69 (0.56-0.84)**
	1.3 < PIR < 3.5	61.65 (58.48-64.81)	53.74 (50.59-56.88)	0.74 (0.60-0.90)*
	PIR ≥ 3.5	67.23 (62.03-72.43)	58.03 (53.75-62.30)	0.65 (0.50-0.86)*
Smoking (%)	PIR ≤ 1.30	18.89 (16.52-21.26)	29.97 (26.73-33.21)	1.84 (1.47-2.30)**
	1.3 < PIR < 3.5	12.34 (10.03-14.64)	21.61 (18.89-24.33)	1.96 (1.51-2.55)**
	PIR ≥ 3.5	11.66 (8.11-15.21)	16.01 (12.64-19.38)	1.44 (0.98-2.12)
Alcohol Consumption (%)	PIR ≤ 1.30	30.46 (27.33-33.60)	46.37 (42.56-50.17)	2.04 (1.64-2.53)**
	1.3 < PIR < 3.5	41.37 (37.43-45.31)	54.07 (50.34-57.80)	1.64 (1.33-2.02)**
	PIR ≥ 3.5	61.47 (56.65-66.28)	69.14 (65.49-72.80)	1.41 (1.07-1.86)*
Insufficient Physical Activity (%)	PIR ≤ 1.30	45.43 (41.57-49.29)	38.07 (34.56-41.58)	0.60 (0.50-0.72)**
	1.3 < PIR < 3.5	41.35 (37.75-44.95)	34.59 (31.13-38.05)	0.62 (0.47-0.82)**
	PIR ≥ 3.5	39.16 (33.59-44.73)	29.68 (25.73-33.64)	0.60 (0.44-0.82)*
Sedentary Behavior (%)	PIR ≤ 1.30	27.01 (23.58-30.45)	28.87 (25.30-32.43)	1.06 (0.87-1.29)
	1.3 < PIR < 3.5	26.38 (23.27-29.49)	28.10 (24.57-31.63)	1.05 (0.82-1.35)
	PIR ≥ 3.5	36.22 (31.15-41.29)	41.00 (36.31-45.68)	1.28 (0.93-1.77)

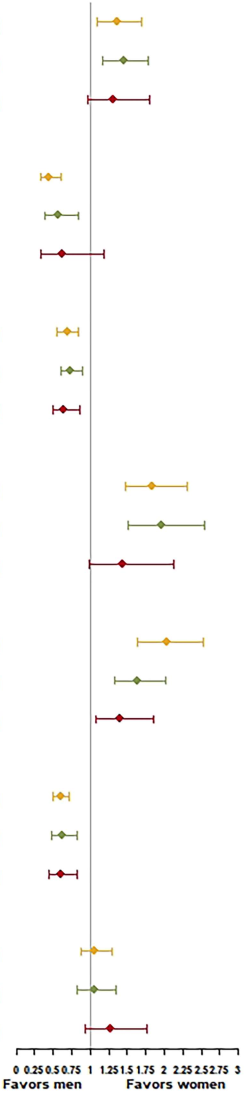

Supplement: Supplementary file 1 [file ijerph-19-16412-s001.zip › ijerph-2023050-supplementary.pdf]
